# If at first you don’t succeed, when should you try again? A prospective study of failed quit attempts and subsequent smoking cessation

**DOI:** 10.1016/j.addbeh.2020.106366

**Published:** 2020-07

**Authors:** Sarah E. Jackson, Robert West, Jamie Brown

**Affiliations:** Department of Behavioural Science and Health, University College London, London, UK

**Keywords:** Smoking cessation, Quit attempts, Quit success

## Abstract

**Objective:**

To assess the association between likelihood of success of smoking cessation attempts and time since most recent attempt.

**Methods:**

Prospective study of 823 smokers who reported a failed quit attempt in the last 12 months at baseline and ≥1 quit attempt over 6-month follow-up. The input variable was time in months between the end (and in an exploratory analysis, the start) of the most recent failed quit attempt reported retrospectively at baseline and start of the first attempt made during the 6-month follow-up period. The outcome variable was success in the latter quit attempt.

**Results:**

Success rates for failed quitters who waited <3, 3–6, and 6–12 months between their failed quit attempt ending and making a subsequent quit attempt were 13.8%, 17.5%, and 19.0% respectively. After adjustment for covariates, the odds of cessation relative to those who made a subsequent quit attempt within 3 months were 1.42 (95%CI 0.79–2.55) and 1.52 (95%CI 0.81–2.86) for those who waited 3–6 and 6–12 months respectively before trying again. Bayes factors indicated the data were insensitive. The exploratory analysis showed the odds of cessation were 1.55 (95%CI 0.78–3.08), 1.92 (95%CI 0.94–3.92), and 2.47 (95%CI 1.04–5.83) greater for those with an interval of 3–6, 6–12, and 12–18 months respectively than those who tried again within 3 months.

**Conclusions:**

While pre-planned analyses were inconclusive, exploratory analysis of retrospective reports of quit attempts and success suggested the likelihood of success of quit attempts may be positively associated with number of months since beginning a prior quit attempt. However, only the longest inter-quit interval examined (12–18 months) was associated with significantly greater odds of quit success relative to a <3 month interval in fully adjusted models; all other comparisons were inconclusive.

## Highlights

• Data were from 823 smokers who had failed in a quit attempt and tried to quit again.
• We analysed success rates in relation to the time interval between quit attempts.
• Of those who waited <3, 3–6 and 6–12 months, 13.8%, 17.5% and 19.0% were successful.
• This difference was not statistically significant, with data proving insensitive.

## 1 Introduction

Tobacco smoking remains one of the leading causes of preventable death worldwide (World Health Organization. Global status report on noncommunicable diseases, 2014). The majority of smokers want to quit (Office of the Surgeon General (US) and Office on Smoking and Health (US), 2004) and many try to quit each year (Hyland et al., 2006, Ahluwalia et al., 2018), but the chance of success of any given quit attempt is low, with fewer than 5% of unaided attempts succeeding for 12 months or more (Panel TU and DG, 2008). Repeated quit attempts are often needed to become a non-smoker (Getsios et al., 2013, Chaiton et al., 2016). However, while recent failed quit attempts are predictive of future quit attempts (Hyland et al., 2006, Zhou et al., 2009, West et al., 2001), they have also been shown to be associated with subsequent relapse (Zhou et al., 2009). This study assessed whether there was evidence that time since a prior quit attempt within a 12 month window was predictive of quit success in a current quit attempt.

Several studies have shown that having made a recent serious quit attempt prior to a subsequent quit attempt is negatively associated with successful quitting. For example, in the ATTEMPT cohort study, smokers who reported a failed quit attempt in the 3 months before baseline had 61% higher odds of relapse from a subsequent quit attempt than those who had not recently tried to quit (Zhou et al., 2009). Similarly, in a prospective survey of UK smokers, those who had made a quit attempt in the 12 months before baseline had 62% lower odds of success in a subsequent quit attempt than those who had not (West, McEwen, Bolling, & Owen, 2001). However, an analysis of the International Tobacco Control (ITC) Four Country Survey failed to find a significant association between past-year quit attempts and quit success (Hyland et al., 2006). The present study aimed to build on this literature by specifically examining the association between the duration of the interval between quit attempts and likelihood of quit success among failed quitters.

From a theoretical perspective, attempting to quit soon after a failed quit attempt may induce mental fatigue and thereby increase the risk of self-regulatory failure. Mental fatigue is a psychobiological state caused by prolonged periods of demanding cognitive activity (Van Cutsem et al., 2017), and has been linked with decreased motivation (Boksem, Meijman, & Lorist, 2006), task disengagement (Hopstaken, van der Linden, Bakker, & Kompier, 2015), and impaired action monitoring (Boksem et al., 2006), all of which may increase the risk of relapse to smoking. Another possibility is that stressful events or environmental exposures that may influence the likelihood of relapse in a quit attempt are more likely to persist over shorter periods of time than longer intervals.

Understanding how time since a failed quit attempt affects subsequent success in quitting has important practical implications regarding advice given to smokers. For every day a person continues to smoke, their life expectancy is reduced by 4–6 h (Doll, Peto, Boreham, & Sutherland, 2004); so it is imperative that smokers are encouraged to quit as soon as they are able. But if trying to stop smoking too soon after a failed quit attempt reduces the chances of long-term success (Zhou et al., 2009), it may be prudent to encourage failed quitters to take a break before making a subsequent quit attempt.

In summary, this study aimed to investigate how long a smoker should wait after a failed quit attempt before initiating another in order to maximise their chance of success. Using prospective data collected over six months from smokers reporting a recent (≤12 months) failed quit attempt at baseline, we addressed the following research questions:

1. To what extent is the time since the most recent failed quit attempt associated with success in a subsequent quit attempt, after adjustment for sociodemographic characteristics and characteristics of the quit attempt?
2. How far is any such association accounted for by baseline level of cigarette addiction, a key predictor of quit success?

## 2 Method

### 2.1 Design

Data were drawn from the ongoing Smoking Toolkit Study, a monthly cross-sectional survey of a representative sample of adults in England designed to provide insights into population-wide influences on smoking and cessation by monitoring trends on a range of variables relating to smoking (Fidler et al., 2011). The study uses a form of random location sampling to select a new sample of approximately 1700 adults aged ≥16 years each month. Participants complete a face‐to‐face computer‐assisted survey with a trained interviewer. In certain waves (due to availability of funding), baseline smokers who consented to re-contact have been followed up by telephone survey 6 months later. Comparisons with national data indicate that key variables such as sociodemographic characteristics and smoking prevalence are nationally representative (Fidler et al., 2011).

For the present study, we used aggregated data from respondents to the survey from November 2006 through March 2008, June 2008 through March 2012, and September 2014 through September 2016, because these are the only waves in which smokers have been invited to participate in a 6-month follow-up.

### 2.2 Study population

We used data from respondents who (i) reported smoking cigarettes daily (“I smoke cigarettes (including hand-rolled) every day”) or occasionally (“I smoke cigarettes (including hand-rolled), but not every day”) in response to the question “Which of the following best applies to you?” at the time of the baseline survey, (ii) reported having made at least one serious attempt to stop smoking in the last 12 months at baseline, (iii) reported having made at least one serious attempt to stop smoking in the last 6 months at 6-month follow-up, (iv) reported a maximum of 12 months between quit attempts, and (v) provided complete data on all covariates.

### 2.3 Measures

#### 2.3.1 Measurement of exposure: time between quit attempts

Recent quit attempts were assessed at baseline with the question: “How many serious attempts to stop smoking have you made in the last 12 months? By serious attempt I mean you decided that you would try to make sure you never smoked again. Please include any attempt that you are currently making, and please include any successful attempt within the last 12 months.” Those who reported at least one quit attempt were coded 1 and those who reported no quit attempts in the last 12 months were coded 0. By definition, all participants included in the analyses were failed quitters on account of being current smokers at baseline.

Time since the most recent quit attempt failed was calculated at baseline from responses to two questions:

1. “How long ago did your most recent serious quit attempt start? By most recent, we mean the last time you tried to quit. (i) in the last week; (ii) more than a week and up to a month; (iii) more than 1 month and up to 2 months; (iv) more than 2 months and up to 3 months; (v) more than 3 months and up to 6 months; (vi) more than 6 months and up to a year.”
2. “How long did your most recent serious quit attempt last before you went back to smoking? (i) less than a day; (ii) less than a week; (iii) more than a week and up to a month; (iv) more than 1 month and up to 2 months; (v) more than 2 months and up to 3 months; (vi) more than 3 months and up to 6 months; (vii) more than 6 months and up to a year.”

Quit attempts were assessed at 6-month follow-up with the question: “How many serious attempts to stop smoking have you made in the last 6 months? By serious attempt I mean you decided that you would try to make sure you never smoked again. Please include any attempt that you are currently making, and please include any successful attempt within the last 6 months.” Those who reported having made between one and three quit attempts were included in the analyses, because information on time since the quit attempt began was only collected for the three most recent quit attempts, and our exposure was calculated based on the interval between the most recent quit attempt at baseline and the first quit attempt that occurred over the follow-up period.

Time since the first quit attempt reported at 6-month follow-up began was assessed with the question: “How long ago did your [most recent/second most recent/third most recent] serious quit attempt start? (i) In the last week; (ii) more than a week and up to a month; (iii) more than 1 month and up to 2 months; (iv) more than 2 months and up to 3 months; (v) more than 3 months and up to 6 months”.

Time between quit attempts was calculated in months as the midpoint of the response category for time since the most recent quit attempt at baseline began minus the midpoint of the response category for how long this quit attempt lasted (to get the time since the most recent quit attempt failed), plus 6 months (follow-up period), minus the midpoint of the response category for time since the first quit attempt reported at 6-month follow-up began. For example, for a participant who reported their most recent failed quit attempt at baseline starting 2–3 months before the baseline survey and lasting 1–2 months, and their first quit attempt in the 6-month follow-up period starting 1–2 months before the follow-up survey, the time between quit attempts was calculated as 2.5 months (midpoint between 2 and 3 months) – 1.5 months (midpoint between 1 and 2 months) + 6 months (follow-up period) – 1.5 months (midpoint between 1 and 2 months) = 5.5 months.

For our primary analysis, time between quit attempts was categorised as <3 months, 3–6 months, and 6–12 months on the basis that a categorical approach to coding the inter-quit interval would allow for more direct translation of the results into recommendations made to smokers (e.g. wait × number of months before trying again) than would a continuous variable (e.g. wait longer before trying again). The reference group was those who had a ≤3 month interval between quit attempts.

#### 2.3.2 Measurement of outcome: quit success

Quit success at 6-month follow-up was assessed with the question: “How long did [your] quit attempt last before you went back to smoking?” Quit success was coded 1 for those who answered “still not smoking” and 0 for those who answered “less than a day”, “less than a week”, “more than 1 week and up to a month”, “more than 1 month and up to 2 months”, “more than 2 months and up to 3 months”, or “more than 3 months and up to 6 months”.

#### 2.3.3 Measurement of covariates

Sociodemographic characteristics, measured at baseline, included: age, sex, and social grade (an occupational index of socioeconomic position (National Readership Survey, 2007).

Variables relating to the first quit attempt reported at 6-month follow-up included: time since the quit attempt began, whether the quit attempt was abrupt or gradual, and use of an evidence-based smoking cessation aid (prescription medication [varenicline, bupropion, nicotine replacement therapy], behavioural support, or e-cigarettes).

Baseline level of cigarette addiction was assessed with strength of urges to smoke (an indicator of addiction that closely predicts relapse in this population (Fidler, Shahab, & West, 2011), rated on a continuous scale from 0 (no urges) to 5 (extremely strong urges).

### 2.4 Statistical analysis

The analysis plan was pre-registered on Open Science Framework (https://osf.io/ht7ue/files/). Analyses were conducted in SPSS v.25 on complete cases.

We analysed associations between covariates and time between quit attempts (≤3 months, 3–6 months, 6–12 months) using Pearson’s chi-square analyses for categorical variables and one-way analysis of variance for continuous variables.

For our primary analyses, we used logistic regression to analyse the association between time between quit attempts and quit success at 6-month follow-up. We constructed four models. Model 1 was unadjusted. Model 2 adjusted for age, sex, social grade, baseline motivation to stop smoking, time since the latter quit attempt began, whether it was abrupt or gradual, and use of an evidence-based cessation aid. Model 3 was fully adjusted for all variables in model 2 and baseline level of cigarette addiction. Model 4 was an addition to our pre-registered models and additionally adjusted for how long the failed quit attempt lasted (entered as a categorical variable based on original response options).

In a planned sensitivity analysis, we repeated the models separating the group with ≤3 months between quit attempts to distinguish between those with <1 month and those with 1–3 months between quit attempts. The reference group was those who had a 1–3 month interval between quit attempts.

In response to inconclusive results from the primary analyses, we also added an unplanned sensitivity analysis in which the time between quit attempts was recalculated as the time from the start (rather than the end) of the failed quit attempt to the start of the subsequent quit attempt. Our rationale was that it is often unclear when a quit attempt ends and participants may not have been able to accurately recall this, introducing noise into our original exposure variable. This was calculated in months as the midpoint of the response category for time since the most recent quit attempt at baseline began, plus 6 months (follow-up period), minus the midpoint of the response category for time since the first quit attempt reported at 6-month follow-up began, and categorised as <3 months, 3–6 months, 6–12 months, and 12–18 months. We reran the first three models from our primary analyses with additional adjustment in Models 2 and 3 for how long the failed quit attempt lasted. The reference group was those who had a <3 month interval between the start of their quit attempts.

Following peer review, we added an additional unplanned sensitivity analysis in which we excluded non-daily smokers from the sample. The rationale for this was that non-daily smokers may quit less frequently than daily smokers but succeed at a higher rate once they do make a quit attempt.

In order to aid interpretation of non-significant associations, we calculated Bayes factors to differentiate between evidence for no effect from data insensitivity. Bayes factors were calculated using an online calculator (http://www.lifesci.sussex.ac.uk/home/Zoltan_Dienes/inference/Bayes.htm) with alternative hypotheses represented by half-normal distributions and the expected effect size set to OR = 1.3 on the basis of previous research into associations between other characteristics of quit attempts and odds of quit success (Garnett, Shahab, Raupach, West, & Brown, 2019). Bayes factors >1 indicate that the data support the alternative hypothesis (in this case, increased odds of quit success with a longer interval between quit attempts) and <1 indicate that the data support the null hypothesis. Bayes factors ≥3 can be interpreted as evidence for the alternative hypothesis (and against the null), ≤1/3 as evidence for the null hypothesis, and between 1/3 and 3 suggest the data are insensitive to distinguish the alternative hypothesis from the null (Dienes, 2014, Jeffreys, 1961).

## 3 Results

A total of 34,440 smokers responded to the baseline Smoking Toolkit Survey in the relevant waves, of whom 11,273 (32.7%) reported at least one failed quit attempt in the preceding 12 months. Follow-up data were available for 2,384 (21.1%) failed quitters, of whom 1037 (43.5%) reported having made between one and three quit attempts during the 6-month follow-up period. We excluded 214 people with missing data, leaving a final sample of 823 participants. The majority of missing values (*n* = 135) were on the time between quit attempts variable, either because people did not report or were not sure when their quit attempts started or how long their initial quit attempt lasted. Of the total eligible sample of failed quitters, those who were retained for analysis were significantly older than those lost to follow-up or excluded on the basis of missing data, included a higher proportion of female and higher social grade smokers, and reported stronger urges to smoke (Table S1). Table 1 summarises characteristics of the analysed sample overall and in relation to time between quit attempts.

**Table 1:** Sample characteristics overall and by time between quit attempts (calculated as the end of the first quit attempt to the start of the subsequent quit attempt).

	Whole sample (*n* = 823)	<3 months (*n* = 159)	3–6 months (*n* = 349)	6–12 months (*n* = 315)	*p*^1^
*Sociodemographic characteristics at baseline*					
Age in years, % (*n*)					
16–24	10.6 (87)	8.2 (13)	9.5 (33)	13.0 (41)	0.502
25–34	16.9 (1 3 9)	18.9 (30)	16.6 (58)	16.2 (51)	–
35–44	22.0 (1 8 1)	20.8 (33)	22.3 (78)	22.2 (70)	–
45–54	20.5 (1 6 9)	26.4 (42)	19.2 (67)	19.0 (60)	–
55–64	17.7 (1 4 6)	14.5 (23)	70 (20.1)	16.8 (53)	–
≥65	12.3 (1 0 1)	11.3 (18)	43 (12.3)	12.7 (40)	–
Female sex, % (*n*)	58.3 (4 8 0)	57.9 (92)	58.7 (2 0 5)	58.1 (1 8 3)	0.977
Social grade, % (*n*)					
AB	13.5 (1 1 1)	16.4 (26)	14.9 (52)	10.5 (33)	0.345
C1	22.1 (1 8 2)	17.0 (27)	21.8 (76)	25.1 (79)	–
C2	21.7 (1 7 9)	24.5 (39)	20.9 (73)	21.3 (67)	–
D	16.4 (1 3 5)	17.0 (27)	17.5 (61)	14.9 (47)	–
E	26.2 (2 1 6)	25.2 (40)	24.9 (87)	28.3 (89)	–

*Characteristics relating to the latter quit attempt*					
Time since quit attempt began, % (*n*)					
≤1 week	5.6 (46)	0.0 (0)a,b,c,, a,b,c,	3.4 (12)a,b,c,, a,b,c,	10.8 (34)a,b,c,, a,b,c,	<0.001
greater than 1–4 weeks	10.2 (84)	0.0 (0)a,b,c,, a,b,c,	9.5 (33)a,b,c,, a,b,c,	16.2 (51)a,b,c,, a,b,c,	–
greater than 1–2 months	13.6 (1 1 2)	1.9 (3)a,b,c,, a,b,c,	13.2 (46)a,b,c,, a,b,c,	20.0 (63)a,b,c,, a,b,c,	–
greater than 2–3 months	22.2 (1 8 3)	1.9 (3)a,b,c,, a,b,c,	26.9 (94)^a^	27.3 (86)^b^	–
greater than 3–6 months	48.4 (3 9 8)	96.2 (1 5 3)a,b,c,, a,b,c,	47.0 (1 6 4)a,b,c,, a,b,c,	25.7 (81)a,b,c,, a,b,c,	–
Quit attempt was abrupt, % (*n*)	53.3 (4 3 9)	55.3 (88)	56.7 (1 9 8)	48.6 (1 5 3)	0.093
Used evidence-based cessation aid, % (*n*)	37.8 (3 1 1)	45.3 (72)	36.4 (1 2 7)	35.6 (1 1 2)	0.093

*Baseline level of cigarette addiction*					
Strength of urges (0–5), mean (SD)	2.27 (1.03)	2.28 (1.00)	2.23 (1.00)	2.32 (1.08)	0.545

1 *p* value for the association between each variable and time between quit attempts.

a,b,c, Where the omnibus *p* value was < 0.05, paired contrasts showed significant differences between groups with matching letters.

Success rates for failed quitters who waited <3 months, 3–6 months, and 6–12 months before making a subsequent quit attempt were 13.8%, 17.5%, and 19.0% respectively. Sequentially adjusted models (Table 2) revealed no significant differences in the odds of quit success in relation to time between quit attempts, and Bayes factors indicated that the data were insensitive to distinguish between a small or no effect.

**Table 2:** Association between time between quit attempts (calculated as the end of the first quit attempt to the start of the subsequent quit attempt) and subsequent quit success.

			Model 1^1^			Model 2^2^			Model 3^3^			Model 4^4^		
Time between quit attempts	*n*	Success rate % (*n*)	OR [95% CI]	*p*	BF^5^	OR [95% CI]	*p*	BF^5^	OR [95% CI]	*p*	BF^5^	OR [95% CI]	*p*	BF^45^
<3 months	159	13.8 (22)	1.00	–	–	1.00	–	–	1.00	–	–	1.00	–	–
3–6 months	349	17.5 (61)	1.32 [0.78–2.24]	0.304	1.20	1.41 [0.79–2.51]	0.252	1.21	1.42 [0.79–2.55]	0.236	1.22	1.57 [0.86–2.67]	0.143	1.34
6–12 months	315	19.0 (60)	1.47 [0.86–2.49]	0.158	1.32	1.47 [0.78–2.74]	0.233	1.21	1.52 [0.81–2.86]	0.192	1.22	1.84 [0.94–3.61]	0.077	1.23

OR, odds ratio. 95% CI, 95% confidence interval. BF, Bayes factor.

1 Unadjusted model.

2 Adjusted for age, sex, social grade, baseline motivation to stop smoking, time since the latter quit attempt began, whether it was abrupt or gradual, and use of an evidence-based cessation aid.

3 Adjusted for all variables in model 2 and baseline level of cigarette addiction.

4 Adjusted for all variables in model 3 and how long the failed quit attempt lasted.

5 Bayes factors ≥ 3 can be interpreted as evidence for the alternative hypothesis (and against the null), ≤1/3 as evidence for the null hypothesis, and between 1/3 and 3 suggest the data are insensitive to distinguish the alternative hypothesis from the null.

When we subdivided the group reporting an interval of <3 months between quit attempts, the success rate was 14.6% for those who waited 1–3 months but 0% for those who waited <1 month. However, there were only 8 participants in the latter group so this result should be interpreted with caution. Because there were no successful quitters in the group of failed quitters who waited <1 month before trying again, it was not possible to calculate their odds of quit success relative to those who waited longer. Odds of quit success for failed quitters who waited 3–6 and 6–12 months relative to those who waited 1–3 months were very similar to those observed when participants who waited < 1 month were included in the reference category (Table S2).

Exploratory analyses based on the time between the start of the failed quit attempt and the start of a subsequent quit attempt (Table 3) included a slightly larger number of participants (*n* = 918) on account of including those with an 12–18 month interval between quit attempts. Quit success rates were 11.7%, 15.3%, 19.9%, and 22.4% for those with an interval of < 3 months, 3–6 months, 6–12 months, and 12–18 months respectively. After full adjustment for covariates (including how long the failed quit attempt lasted), odds of quit success were significantly higher for failed quitters who had an interval of 12–18 months between the start of consecutive quit attempts than those who had an interval of < 3 months (Model 3, Table 3). Differences between those with an interval of 3–6 or 6–12 months and those with an interval of < 3 months were not statistically significant, with data proving insensitive.

**Table 3:** Association between time between quit attempts (calculated as the start of first quit attempt to the start of the subsequent quit attempt) and quit success.

			Model 1^1^			Model 2^2^			Model 3^3^		
Time between quit attempts	*n*	Success rate % (*n*)	OR [95% CI]	*p*	BF^4^	OR [95% CI]	*p*	BF^4^	OR [95% CI]	*p*	BF^4^
<3 months	120	11.7 (14)	1.00	–		1.00	–		1.00	–	
3–6 months	300	15.3 (46)	1.37 [0.72–2.60]	0.333	1.14	1.53 [0.77–3.05]	0.224	1.18	1.55 [0.78–3.08]	0.216	1.18
6–12 months	306	19.9 (61)	1.89 [1.01–3.52]	0.046	–	1.79 [0.88–3.65]	0.108	1.19	1.92 [0.94–3.92]	0.075	1.20
12–18 months	192	22.4 (43)	2.19 [1.14–4.20]	0.019	–	2.36 [1.00–5.57]	0.050	1.11	2.47 [1.04–5.83]	0.040	–

OR, odds ratio. 95% CI, 95% confidence interval. BF, Bayes factor.

1 Unadjusted model.

2 Partially adjusted model, including age, sex, social grade, baseline motivation to stop smoking, how long the failed quit attempt lasted, time since the latter quit attempt began, whether it was abrupt or gradual, and use of an evidence-based cessation aid.

3 Fully adjusted model, including all variables in model 2 and baseline level of cigarette addiction.

4 Bayes factors were only calculated for non-significant results. Bayes factors ≥ 3 can be interpreted as evidence for the alternative hypothesis (and against the null), ≤1/3 as evidence for the null hypothesis, and between 1/3 and 3 suggest the data are insensitive to distinguish the alternative hypothesis from the null.

Excluding non-daily smokers from the sample did not substantially alter the pattern of results (Tables S3 and S4).

## 4 Discussion

The results showed that a longer interval between quit attempts was associated with a small but non-significant increase in success rates, while Bayes factors indicated that the data were insensitive to distinguish between a small effect or no effect (i.e., the null hypothesis of no association between the duration of the inter-quit interval and quit success could not be ruled out). Exploratory analyses in which the time between quit attempts was calculated as the duration between the start of the failed quit attempt and the start of the next quit attempt (with adjustment for the duration of the failed quit attempt) found that failed quitters who reported intervals of 3–6 months, 6–12 months, and 12–18 months between the start of their quit attempts had approximately 1.5, 2.0, and 2.5 times higher odds respectively of reporting continuous abstinence up to 6 months later than those who reported an interval of < 3 months. The difference between those who waited 12–18 months and those who waited < 3 months was statistically significant, but data were insensitive for comparisons between the 3–6 and 6–12 month groups and the < 3 month group.

Our mixed findings reflect the inconsistencies in the existing literature. The failure to observe a significant difference in our planned analyses is in line with results of the ITC Four Country Survey, which suggested that the odds of quit success did not differ significantly between smokers who had made a quit attempt in the past year versus those who had not (Hyland et al., 2006). However, the exploratory results provide support for previous studies that have shown a higher rate of relapse among smokers who reported a failed quit attempt in the 3 months (Zhou et al., 2009) or 12 months (West et al., 2001) prior to the start of a subsequent quit attempt.

A reduction in likelihood of success when trying to quit soon after a failed quit attempt could be due to a range of factors. One is insufficient time to recover from ‘mental fatigue’. Mental fatigue is likely to result from a period of attempting to stop smoking, which many people find extremely demanding. Without sufficient time to recover, this fatigue may increase likelihood of subsequent relapse by decreased motivation (Boksem et al., 2006), task disengagement (Hopstaken et al., 2015), and impaired action monitoring (Boksem et al., 2006). Another possibility is that stressful events or environmental exposures that can precipitate a relapse – such as changes in employment or relationships, deaths, or financial crisis (McCabe, Cranford, & Boyd, 2016) – are more likely to persist over shorter periods of time than longer intervals. Finally, another possible factor may be a loss of self-efficacy (Gwaltney, Metrik, Kahler, & Shiffman, 2009).

While we adjusted our analyses for sociodemographic characteristics, level of cigarette addiction, and features of the quit attempt that may influence the odds of successful cessation, it is possible that the results are confounded by unmeasured variables. For example, it could be that smokers in the sample who were attempting to quit more frequently were those who had external factors pushing them to stop smoking (e.g. health problems, financial concerns) but a lack of support to succeed. Studies that have collected information daily from smokers who intended to stop smoking have documented substantial variability in changes in smoking behaviour between individuals (Hughes et al., 2013, Hughes et al., 2014). While some smokers plan their quit attempts in advance, many (if not most) quit attempts are not carefully planned (Hughes et al., 2013, Hughes et al., 2014). The majority of quit attempts last less than a day (Hughes et al., 2013, Hughes et al., 2014). Our finding that time between quit attempts may moderate success may correspond to other factors that self-select poorer outcomes for smokers who seem to quit often without success.

There is a need for further research – ideally experimental, to overcome issues related to confounding and causal inference inherent in observational studies and to achieve more precise measurement of the inter-quit interval – in order to draw firm conclusions as to whether waiting longer after a failed quit attempt before trying again increases the chances of successful cessation. This could be tested by randomising smokers receiving behavioural support to wait for different lengths of time after a failed quit attempt before trying again. The interaction between inter-quit interval and use of different cessation aids could also be explored. Should a positive association between time since a failed quit attempt and success in a subsequent quit attempt be observed, there may be important implications for the advice given to smokers who want to quit. In gauging the optimal time interval between quit attempts, it will be important to weigh the benefits of leaving a longer interval between quit attempts (to increase the chance of successful cessation) against the increased health risks associated with continuing to smoke for even slightly longer than necessary (Doll et al., 2004). While the aim should be to try and maximise the chance of success in each quit attempt, it is important to consider that the majority of smokers who attempt to quit are unsuccessful – even when they wait more than a year before trying again, as is evident from our results. Rather than advising people motivated to quit smoking that it might be better to wait longer following an unsuccessful quit attempt, suggesting that having sufficient time to adequately prepare for the next quit attempt might be advantageous. Further research using simulation modelling could provide insight into the time interval that minimises risk to health. We also need to understand which other variables influence the relationship between time between quit attempts and quit success, and to what extent, in order to understand what treatments might be useful in helping failed quitters achieve abstinences.

Alternative treatment approaches that could maximise the chances of successful cessation soon after a failed quit attempt include stepped care, providing counselling that targets the relapse risk factors suspected of triggering the most recent relapse, contingency management interventions (which incentivise behaviour change via the offer of rewards (Notley et al., 2019)), or harm reduction approaches (e.g. substituting some cigarettes for use of an e-cigarette or nicotine replacement product) that carry less risk than continuing to smoke.

This study had several limitations. Our (planned) measure of time between quit attempts was calculated based on responses to three self-reported, recall-based measures (time since the failed quit attempt started, how long it lasted, and time since the first quit attempt reported at follow-up started), introducing scope for bias. It is possible that participants were able to recall making a quit attempt but were not able to accurately recall the timing of this quit attempt or how long it lasted for, which could at least partly account for the null findings in our planned analyses. Bias may also have been introduced by participants forgetting failed quit attempts, particularly if short duration attempts that occurred longer ago were more likely to be forgotten or perceived as not serious. In addition, we did not have precise information on the timing and duration of quit attempts, so our calculation of time between quit attempts relied on using the midpoint of ranges provided in the response options. While some of these ranges were quite narrow (e.g. less than a week), others were relatively wide (e.g. 6–12 months) which will have reduced the accuracy of our estimates. Inclusion of how long the failed quit attempt lasted in our measure of time between quit attempts may have introduced noise because it is often unclear when a quit attempt ends which may have reduced participants’ ability to accurately report this. In the unplanned sensitivity analysis that removed this variable (based on midpoints of response options) from the calculation of time between quit attempts but included it (as a categorical variable based on the original response options) as a covariate, there was some evidence that a longer interval between quit attempts was associated with greater odds of quit success. It is possible that recalculating our exposure based on two rather than three variables reduced the level of noise, or that using the end of the quit attempt rather than the start in our planned analyses was confounding two factors working in opposite directions (i.e. the recency of the failed quit attempt and the length of time the quit attempt succeeded for). Finally, there was considerable loss to follow-up with differences on key sociodemographic characteristics and level of addiction between the analysed sample and those who were excluded, which may reduce the extent to which our results are representative of failed quitters in England.

In conclusion, exploratory analysis of retrospective reports of quit attempts and quit success suggests that the likelihood of success of quit attempts may be positively associated with number of months since a preceding quit attempt. However, only the longest inter-quit interval examined (12–18 months) was associated with significantly greater odds of quit success relative to a < 3 month interval in fully adjusted models, while all other comparisons were inconclusive.

## 5 Ethics approval and consent to participate

Ethical approval for the STS was granted originally by the UCL Ethics Committee (ID 0498/001). The data are not collected by UCL and are anonymized when received by UCL.

## 6 Availability of data and materials

Data are available from the authors upon reasonable request.

## Author contributions

SEJ, RW and JB designed the study. SEJ analysed the data and drafted the manuscript. RW and JB made critical revisions. All authors read and approved the final version.

## Funding

Cancer Research UK funded the Smoking Toolkit Study data collection (C1417/A22962; C44576/A19501) and SJ & JB’s salary (C1417/A22962). The funders had no final role in the study design; in the collection, analysis and interpretation of data; in the writing of the report; or in the decision to submit the paper for publication. All researchers listed as authors are independent from the funders and all final decisions about the research were taken by the investigators and were unrestricted. All authors had full access to all of the data (including statistical reports and tables) in the study and can take responsibility for the integrity of the data and the accuracy of the data analysis.

## CRediT authorship contribution statement

**Sarah E. Jackson:** Conceptualization, Formal analysis, Investigation, Methodology, Writing - original draft, Writing - review & editing. **Robert West:** Conceptualization, Data curation, Funding acquisition, Investigation, Methodology, Resources, Supervision, Writing - review & editing. **Jamie Brown:** Conceptualization, Data curation, Funding acquisition, Investigation, Methodology, Resources, Supervision, Writing - review & editing.

## Declaration of Competing Interest

JB has received unrestricted research funding to study smoking cessation from Pfizer, who manufacture smoking cessation medications. RW undertakes research and consultancy for and receives travel funds and hospitality from manufacturers of smoking cessation medications (Pfizer, GlaxoSmithKline and Johnson and Johnson). All authors declare no financial links with tobacco companies or e-cigarette manufacturers or their representatives.
