# Supplementary material for: If at first you don’t succeed, when should you try again? A prospective study of failed quit attempts and subsequent smoking cessation
Source: Addict Behav. 2020 Jul;106:106366. doi: 10.1016/j.addbeh.2020.106366 (PMC7163297; doi:10.1016/j.addbeh.2020.106366)
Supplement: Supplementary data 1 [file mmc1.docx]

| **Table S1.** Characteristics of the analysed sample compared with all failed quitters at baseline | | | | | | | |  |  |  |
| --- | --- | --- | --- | --- | --- | --- | --- | --- | --- | --- |
|  |  | | **Analysed sample**  **(*n*=823)** | **All failed quitters (*n*=10451)** | ***p*^1^** | |  |  |  |  |
| **Sociodemographic characteristics at baseline** | | |  |  |  | |  |  |  |  |
|  | Age in years, % (*n*) | |  |  |  | |  |  |  |  |
|  |  | 16-24 | 10.6 (87) | 21.8 (2274) | <0.001 | |  |  |  |  |
|  |  | 25-34 | 16.9 (139) | 24.1 (2523) | - | |  |  |  |  |
|  |  | 35-44 | 22.0 (181) | 20.4 (2136) | - | |  |  |  |  |
|  |  | 45-54 | 20.5 (169) | 15.6 (1627) | - | |  |  |  |  |
|  |  | 55-64 | 17.7 (146) | 10.8 (1130) | - | |  |  |  |  |
|  |  | ≥65 | 12.3 (101) | 7.3 (761) | - | |  |  |  |  |
|  | Female sex, % (*n*) | | 58.3 (480) | 53.4 (5577) | 0.006 | |  |  |  |  |
|  | Social grade, % (*n*) | |  |  |  | |  |  |  |  |
|  |  | AB | 13.5 (111) | 10.3 (1080) | 0.016 | |  |  |  |  |
|  |  | C1 | 22.1 (182) | 22.8 (2381) | - | |  |  |  |  |
|  |  | C2 | 21.7 (179) | 23.0 (2402) | - | |  |  |  |  |
|  |  | D | 16.4 (135) | 19.3 (2020) | - | |  |  |  |  |
|  |  | E | 26.2 (216) | 24.6 (2568) | - | |  |  |  |  |
| **Baseline level of cigarette addiction** | | |  |  |  | | | |  |  |
|  | Strength of urges (0-5), mean (SD) | | 2.27 (1.03) | 2.20 (1.05) | | 0.046 | |  |  |  |
| ^1^ *p* value for the difference between the analysed sample and those excluded from the analyses because of loss to follow-up or missing data. | | | | | | | |  |  |  |

| **Table S2.** Association between time between quit attempts and subsequent quit success: sensitivity analysis | | | | | | | | | | | | | | |
| --- | --- | --- | --- | --- | --- | --- | --- | --- | --- | --- | --- | --- | --- | --- |
|  |  |  | **Model 1^1^** | |  |  | | **Model 2^2^** | |  |  | **Model 3^3^** | |  |
| **Time between quit attempts** | ***n*** | **Success rate  % (*n*)** | **OR [95% CI]** | ***p*** | **BF^4^** |  | **OR [95% CI]** | | ***p*** | **BF^4^** |  | **OR [95% CI]** | ***p*** | **BF^4^** |
| <1 month | 8 | 0.0 (0) | -^5^ | - |  |  | -^5^ | |  |  |  | -^5^ | - |  |
| 1-3 months | 151 | 14.6 (22) | 1.00 | - |  |  | 1.00 | | - |  |  | 1.00 | - |  |
| 3-6 months | 249 | 17.5 (61) | 1.24 [0.73-2.11] | 0.423 | 1.11 |  | 1.31 [0.73-2.35] | | 0.358 | 1.14 |  | 1.33 [0.74-2.39] | 0.341 | 1.16 |
| 6-12 months | 315 | 19.0 (60) | 1.38 [0.81-2.35] | 0.236 | 1.24 |  | 1.37 [0.73-2.58] | | 0.324 | 1.15 |  | 1.42 [0.76-2.68] | 0.274 | 1.18 |
| OR, odds ratio. 95% CI, 95% confidence interval.  ^1^ Unadjusted model.  ^2^ Partially adjusted model, including age, sex, social grade, baseline motivation to stop smoking, time since the latter quit attempt began, whether it was abrupt or gradual, and use of an evidence-based cessation aid.  ^3^ Fully adjusted model, including all variables in model 2 and baseline level of cigarette addiction.  ^4^ Bayes factors ≥3 can be interpreted as evidence for the alternative hypothesis (and against the null), ≤1/3 as evidence for the null hypothesis, and between 1/3 and 3 suggest the data are insensitive to distinguish the alternative hypothesis from the null.  ^5^ Odds ratios and 95% confidence intervals could not be computed for those with ≤1 month between quit attempts because there were no successful quitters in this group. | | | | | | | | | | | | | | |

| **Table S3.** Association between time between quit attempts (calculated as the end of the first quit attempt to the start of the subsequent quit attempt) and subsequent quit success: daily smokers only | | | | | | | | | | | | | | | | | | |
| --- | --- | --- | --- | --- | --- | --- | --- | --- | --- | --- | --- | --- | --- | --- | --- | --- | --- | --- |
|  |  |  | **Model 1^1^** | |  |  | | **Model 2^2^** | |  |  | **Model 3^3^** | | |  | **Model 4^4^** | | |
| **Time between quit attempts** | ***n*** | **Success rate  % (*n*)** | **OR  [95% CI]** | ***p*** | **BF^5^** |  | **OR**  **[95% CI]** | | ***p*** | **BF^5^** |  | **OR**  **[95% CI]** | ***p*** | **BF^5^** |  | **OR**  **[95% CI]** | ***p*** | **BF^45^** |
| <3 months | 136 | 11.8 (16) | 1.00 | - | - |  | 1.00 | | - | - |  | 1.00 | - | - |  | 1.00 | - | - |
| 3-6 months | 306 | 17.6 (54) | 1.61  [0.88-2.93] | 0.120 | 1.29 |  | 1.62  [0.84-3.12] | | 0.147 | 1.22 |  | 1.63  [0.85-3.14] | 0.142 | 1.23 |  | 1.71  [0.88-3.34] | 0.116 | 1.22 |
| 6-12 months | 290 | 20.0 (58) | 1.88  [1.03-3.40] | 0.039 | - |  | 1.77  [0.89-3.55] | | 0.105 | 1.21 |  | 1.84  [0.92-3.68] | 0.087 | 1.22 |  | 2.05  [0.99-4.27] | 0.055 | 1.18 |
| OR, odds ratio. 95% CI, 95% confidence interval. BF, Bayes factor.  ^1^ Unadjusted model.  ^2^ Adjusted for age, sex, social grade, baseline motivation to stop smoking, time since the latter quit attempt began, whether it was abrupt or gradual, and use of an evidence-based cessation aid.  ^3^ Adjusted for all variables in model 2 and baseline level of cigarette addiction.  ^4^ Adjusted for all variables in model 3 and how long the failed quit attempt lasted.  ^5^ Bayes factors ≥3 can be interpreted as evidence for the alternative hypothesis (and against the null), ≤1/3 as evidence for the null hypothesis, and between 1/3 and 3 suggest the data are insensitive to distinguish the alternative hypothesis from the null. | | | | | | | | | | | | | | | | | | |

| **Table S4.** Association between time between quit attempts (calculated as the start of first quit attempt to the start of the subsequent quit attempt) and quit success: daily smokers only | | | | | | | | | | | | | | | | |
| --- | --- | --- | --- | --- | --- | --- | --- | --- | --- | --- | --- | --- | --- | --- | --- | --- |
|  |  |  | **Model 1^1^** | |  |  | | **Model 2^2^** | |  |  |  | **Model 3^3^** | | | |
| **Time between quit attempts** | ***n*** | **Success rate  % (*n*)** | **OR [95% CI]** | ***p*** | **BF^4^** |  | **OR [95% CI]** | | ***p*** | **BF^4^** |  | **OR [95% CI]** | | ***p*** | **BF^4^** |  |
| <3 months | 105 | 10.5 (11) | 1.00 | - |  |  | 1.00 | | - |  |  | 1.00 | | - |  |  |
| 3-6 months | 262 | 15.6 (41) | 1.59 [0.78-3.22] | 0.202 | 1.18 |  | 1.67 [0.78-3.57] | | 0.187 | 1.16 |  | 1.68 [0.78-3.59] | | 0.184 | 1.16 |  |
| 6-12 months | 276 | 20.3 (56) | 2.18 [1.09-4.34] | 0.027 | - |  | 1.93 [0.88-4.22] | | 0.099 | 1.16 |  | 2.06 [0.94-4.53] | | 0.071 | 1.15 |  |
| 12-18 months | 180 | 22.2 (40) | 2.44 [1.19-5.00] | 0.015 | - |  | 2.37 [0.94-5.97] | | 0.066 | 1.09 |  | 2.45 [0.97-6.17] | | 0.058 | 1.09 |  |
| OR, odds ratio. 95% CI, 95% confidence interval. BF, Bayes factor.  ^1^ Unadjusted model.  ^2^ Partially adjusted model, including age, sex, social grade, baseline motivation to stop smoking, how long the failed quit attempt lasted, time since the latter quit attempt began, whether it was abrupt or gradual, and use of an evidence-based cessation aid.  ^3^ Fully adjusted model, including all variables in model 2 and baseline level of cigarette addiction.  ^4^ Bayes factors were only calculated for non-significant results. Bayes factors ≥3 can be interpreted as evidence for the alternative hypothesis (and against the null), ≤1/3 as evidence for the null hypothesis, and between 1/3 and 3 suggest the data are insensitive to distinguish the alternative hypothesis from the null. | | | | | | | | | | | | | | | | |
